# Optimal Cutoffs for the Diagnosis of Sarcopenia in Older Chinese Adults

**DOI:** 10.3389/fnut.2022.853323

**Published:** 2022-07-05

**Authors:** Sheng Ge, Qin Du, Xiaohui Feng, Yan Liu, Hui Wang, Shan Hai, Xiaodong Shi, Wenguang Sun, Aiqin Ma, Tingting Lv, Haili Liu, Venkata Saibaba Pinupa, Menaka Yalawar, Geraldine E. Baggs, Birong Dong, Wei Chen

**Affiliations:** ^1^Clinical Nutrition Department, Sixth People's Hospital Affiliated Shanghai Jiao Tong University, Shanghai, China; ^2^Department of Clinical Nutrition Science, Abbott Nutrition Research and Development, Shanghai, China; ^3^Peking Union Medical College Hospital, Chinese Academy of Medical Sciences and Peking Union Medical College, Beijing, China; ^4^The Center of Gerontology and Geriatrics, West China Hospital, Sichuan University, Chengdu, China; ^5^Cognizant Technology Solutions, Hyderabad, India; ^6^Cognizant Technology Solutions, Bangalore, India; ^7^Department of Statistical Sciences, Abbott Nutrition Research and Development, Columbus, OH, United States

**Keywords:** sarcopenia, cutoff thresholds, Chinese, older people, dual-energy X-ray absorptiometry, bioelectrical impedance analysis

## Abstract

**Background:**

The optimal criteria for sarcopenia in the older Chinese population have not been defined. Consequently, this study aims to determine the optimal cutoffs of grip strength, appendicular skeletal muscle index (ASMI) using bioelectrical impedance analysis (BIA), and gait speed, comprising the best definition of sarcopenia for older Chinese populations.

**Methods:**

A total of 2,821 (1,398 men and 1,423 women) community-dwelling older people (≥60 years) and 409 (205 men and 204 women) young healthy adults (25–34 years) were recruited from three big cities in China. Besides gait speed and grip strength, we examined ASMI by BIA and dual-energy X-ray absorptiometry (DXA), comprising the three components of sarcopenia. DXA classification for low ASMI, 20th percentile among older adults in the study sample, was found to be best compared with the other existing classification, 1 SD and 2 SD below the mean for the young population, and was used as the gold standard to determine the optimal cutoffs of BIA using receiver operating characteristic curves (ROC). The cutoffs of handgrip strength and gait speed were determined following the same rule.

**Results:**

Using gender-specific 20th percentiles of DXA (6.53 kg/m^2^ for men and 5.40 kg/m^2^ for women), the cutoffs 7.05 kg/m^2^ for men and 5.85 kg/m^2^ for women were determined as optimal cutoffs of BIA by achieving the largest sensitivity (0.81, 95% CI: 0.63–0.93 for men and 0.90, 95% CI: 0.73–0.98 for women) and specificity greater than 0.80 (0.80, 95% CI: 0.72–0.87 for men and 0.81, 95% CI: 0.72–0.87 for women) in the ROC analysis. The 28.5 kg and 1.05 m/s for men and 18.6 kg and 1.01 m/s for women were determined as the cutoffs for handgrip strength and gait speed, respectively. Based on the derived cutoffs, 14.2% of men and 15.7% of women in the older Chinese study population were classified as sarcopenia.

**Conclusion:**

Notably, 7.05 kg/m^2^, 28.5 kg, and 1.05 m/s for men and 5.85 kg/m^2^, 18.6 kg, and 1.01 m/s for women were selected as the optimal cutoffs for low ASMI by BIA, handgrip strength, and gait speed, respectively. These optimal cutoffs will enhance practicability for screening sarcopenia in primary care and clinical settings.

## Introduction

Sarcopenia is a well-known geriatric syndrome characterized by loss of skeletal muscle mass with subsequent low muscle strength (1), leading to impaired health with mobility disorders, increased fall and fracture risk, functional decline, frailty, poor quality of life, and even increased mortality risk (2–5).

Sarcopenia was first described in the 1980s as an age-related decline in lean body mass, affecting mobility, nutritional status, and independence (6). However, since skeletal muscle is influenced by factors, such as race, environment, diet, and exercise habits, sarcopenia still lacks internationally agreed diagnostic criteria, although there are a number of consensus diagnoses. The most widely used of these include the European Working Group on Sarcopenia in Older People (EWGSOP), the International Working Group on Sarcopenia (IWGS), and the Asian Working Group on Sarcopenia (AWGS). Notably, the 2011 IWGS consensus on sarcopenia defines sarcopenia only in terms of a reduction in muscle mass and physical activity (7). In contrast, the EWGSOP consensus published in 2010 considered sarcopenia as a syndrome with adverse consequences, such as functional impairment, reduced quality of life, and even death due to progressive and widespread loss of skeletal muscle mass and strength, recommended the use of tests for muscle mass, muscle strength, and physical performance to diagnose sarcopenia, and classified sarcopenia into pre-muscular hyposmia, sarcopenic phase, and severe sarcopenic phase (8). The Foundation for the National Institute of Health (FNIH) Sarcopenia Project defined sarcopenia in 2014 as clinically relevant low muscle strength (weakness) and low lean mass (9). In 2019, the European Working Group on Sarcopenia in Older People (EWGSOP) summarized the clinical and scientific findings of sarcopenia over the past decade and revised the consensus (EWGSOP2), emphasizing low muscle strength as the primary parameter in the evaluation of sarcopenia, stating that muscle strength is a better predictor of poor outcomes than muscle mass. It was noted that muscle strength is a better predictor of poor disease outcomes than muscle mass (10). The Asian Working Group for Sarcopenia (AWGS) guideline also addressed the cutoff points for older Asian people by modifying the EWGSOP guideline (11) and updated as AWGS2 (12) with a cutoff definition and a new definition of possible sarcopenia in 2019. However, mainland Chinese were not included in the study population in both versions of the AWGS consensus, and the representativeness and application of the definition may be limited in both versions. The prevalence of sarcopenia in mainland China could be inaccurately estimated using the AWGS cutoff values.

The reported prevalence of sarcopenia varies widely depending on its definition. Muscle mass measurement in mainland China remains under-investigated and inadequately validated. Limited studies with small sample sizes or limited geographic diversity have proposed cutoff values for low muscle mass and investigated the prevalence of sarcopenia in mainland China (13, 14). Therefore, developing cutoff values based on the local reference population is essential to better understand the current prevalence of sarcopenia in China.

Moreover, muscle mass can be measured by various techniques, such as magnetic resonance imaging (MRI), computed tomography (CT), dual-energy X-ray absorptiometry (DXA), and bioelectrical impedance analysis (BIA) (15, 16). Considering cost, portability, and technical skills, MRI and CT are more suitable in clinical settings. Moreover, DXA and BIA are two frequently used methods for population studies, and DXA has been recommended as the preferred detection technology for assessing skeletal muscle loss due to its low radiation, accuracy, and strong clinical feasibility (9, 12, 17). BIA is a more portable, easy-to-use, and inexpensive tool compared with DXA. Emerging evidence supports that BIA is valid in the estimation of body composition using DXA as a reference standard (18). However, these validation studies were not conducted on the Chinese population.

Three components of sarcopenia include a low muscle mass (component 1), a low handgrip strength (component 2), and/or a low gait speed (component 3) according to the AWGS recommendation (11). The optimal criteria for sarcopenia in the older Chinese population have not been defined. Consequently, this study aimed to determine the optimal cutoffs of grip strength, appendicular skeletal muscle index (ASMI) using BIA, and gait speed, comprising the best definition of sarcopenia for older Chinese populations.

## Methods

### Study Population

This was a large, multicenter cross-sectional study conducted in 2014–2015 designed to select a representative sample of older Chinese adults (aged ≥60 years) living in urban areas of China to understand the prevalence of sarcopenia in older Chinese adults and also to study the distribution of muscle mass and muscle function in young Chinese adults (aged 25–34 years) for the new criteria of sarcopenia in Chinese people. In brief, this study selected three geographic representative cities (Beijing, Shanghai, and Chengdu) according to the characteristics of the typical life and dietary pattern. After the statistics of communities with similar size and population density in the three cities, one community was selected in each of the three cities according to the random sampling method for sample recruitment. In total, 2,834 older subjects (1,405 men and 1,429 women) participated with a balance in gender. In addition, the number of subjects aged between 60 and 64 years should not exceed one-third of the total target population in each site to match the national population distribution in China. Subjects aged ≥60 years living in the community for more than 12 months with no history of the listed diseases (implanted electronic device or orthopedic metal implantations, hyperthyroidism or hypothyroidism, severe heart dysfunction, renal dysfunction with significant sodium water retention or edema, diuretics intake, and later stage of malignancies with cachexia) were selected as the older reference group.

In addition, 423 young subjects (213 men and 210 women) were recruited from Beijing and Shanghai. The young subjects were healthy men and women aged 25–34 years, had a BMI between 18.5 and 26.9, and with no history of the listed diseases (diabetes, thyroid diseases, cardiovascular diseases, liver diseases, renal diseases, and active infection of tuberculosis, human immunodeficiency virus, and hepatitis). Subjects who were heavy manual laborers, professional or semiprofessional athletes, and with physical disabilities were excluded from both groups.

This study was approved by the Ethics Committee of the institute at each study site (Peking Union Medical College Hospital, The Sixth People's Hospital Affiliated Shanghai Jiaotong University, and West China Hospital Affiliated to Sichuan University). Written informed consent was obtained from each participant. This study was registered at www.clinicaltrials.gov (NCT02089971 and NCT02089906).

### Definitions and Measurements

#### Sarcopenia

Sarcopenia has been described as an age-related decline in skeletal muscle mass and muscle function (defined by muscle strength or physical performance). The recommended diagnostic algorithm of the AWGS, which includes three components, was applied to assess sarcopenia in this study. Study participants with a low muscle mass (component 1), in which DXA was regarded generally as the gold standard measure, plus a low handgrip strength (component 2) and/or a low gait speed (component 3), were regarded as having sarcopenia according to the AWGS recommendation (11, 12).

#### Measurements

Anthropometric measurements were examined by standard protocols and instruments. The subjects wore lightweight clothing without shoes. Bodyweight and height were measured to the nearest 0.1 kg and 0.1 cm, respectively, on a calibrated scale. Calf circumference was measured at the widest point of the participants' legs using a nonelastic measuring tape.

Appendicular skeletal muscle mass (ASM) was measured using two methods, the BIA method using a calibrated bioelectrical impedance analyzer (Inbody 720, Biospace Co., Ltd, Seoul, Korea) and the DXA method using the DXA scanner (Lunar Prodigy, GE Healthcare, Madison, WI, USA) as previously published (18, 19). Both measurements provided the lean body mass of limbs and trunk, body fat mass, and bone mass for all subjects of the young reference group. All eligible subjects of the older group received body composition measurements by BIA. Approximately 10% of the subjects (290 subjects) were randomly selected to receive a muscle mass measurement using the DXA method. ASM was calculated as the sum of the lean body mass of the limbs, which is a good proxy for whole-body skeletal muscle mass (13). ASMI was calculated as ASMI = ASM/height^2^ (kg/m^2^).

There are three existing classifications for defining low ASMI based on DXA: first, ASMI below 2 standard deviations (SDs) from the mean of the young population, second, ASMI below 1 SD from the mean of the young population, and the third, ASMI below 20th percentile of the older population (20–25). We compared the three classifications based on the young study sample to define prevalence in the older study sample. The three cutoff points were used to calculate the rate of low skeletal muscle mass in the older Chinese population for identifying the optimal cutoffs by determining a low muscle mass prevalence between 10 and 40% (2, 22, 26–30) and without gender difference. Based on the lowest 20% of older references, 6.53 and 5.40 kg/m^2^ in men and women were selected as low ASMI cutoff points.

Muscle function was assessed by measuring handgrip strength and gait speed (13). Muscle grip strength was assessed as the best of three attempts with the dominant hand using a squeezing electronic hand dynamometer (Camry EH101, Zhongshan Camry Electronic Co. Ltd, Guangdong, China). Gait speed was assessed as the average value of two tests using the 6-m walk test at self-selected speed by a trained assessor (31). Following the optimum cutoffs for ASMI, we used the 20th percentiles in the older study sample to define low handgrip strength and low gait speed.

All examinations are performed by uniformly trained clinicians with more than 5 years of clinical trial experience in this field. After training, they are tested to make sure that the results they test are repeatable.

### Statistical Analysis

All data analyses were performed using SAS 9.4 software (SAS Institute Inc., Cary, NC, USA). The two-tailed significance level was set to *p* < 0.05. Considering a relatively low prevalence of 5% and 2% as the maximum tolerable error, a total of 2,988 participants with 10% attrition were planned to be enrolled in this study such that we have at least 2,736 evaluable subjects (456 in each center and gender group). In total, 2,834 (1,405 men and 1,429 women) older participants from three centers were enrolled and were stratified by gender and then by age group (60–64 and ≥65). To develop population-specific reference values for low muscle mass using both BIA and DEXA methods, a total of 400 healthy young Chinese participants were enrolled, 100 participants as recommended in the literature for constructing reference intervals for each gender and two centers.

Demographic characteristics, body measurements, and muscle function measurements were presented as mean and SD for continuous variables with normal distribution and absolute numbers and percentages for enumeration data. Gender-specific prevalence of sarcopenia in the older group was calculated according to six existing cutoff values based on the current guidelines of the FNIH, AWGS (and revision), EWGSOP (and revision), and IWGS (7–12). The prevalence of sarcopenia between genders was compared using the chi-square test or Fisher's exact test. The prevalence of low muscle mass and sarcopenia between BIA and DXA in the same individuals who had both measurements was compared using McNemar's test. A simple linear regression and Bland–Altman analysis were conducted to evaluate the agreement between the BIA and DXA result measurements.

To identify the best cutoffs of BIA in two genders, the receiver operating characteristic (ROC) curve analysis was applied using DXA classification of low ASMI as the gold standard. The area under the receiver operating curves was used to determine the optimal cutoffs of BIA in discriminating low ASMI by achieving maximum sensitivity and specificity greater than 0.8. Sensitivity, specificity, positive and negative predictive values, area under the curve (AUC), accuracy between existing guidelines, and our proposed cutoffs were calculated.

## Results

### Participants' Characteristics

A total of 2,821 (1,398 men and 1,423 women) older adults aged ≥60 years and 409 (205 men and 204 women) young adults aged 25–34 years were included in this study, and their characteristics are presented by gender (Table 1).

**Table 1:** Characteristics of the young reference group and older participants by gender.

**Parameters**	**Male**		**Female**	
	**Young sample (*n* = 205)**	**Older sample (*n* = 1,398)**	**Young sample (*n* = 204)**	**Older sample (n = 1,423)**
Age (years)	27.4, 2.7	68.6, 6.3	27.5, 2.8	68.1, 6.0
Height (cm)	172.3, 5.7	165.7, 6.2	160.8, 5.0	154.0, 5.9
Weight (kg)	67.6, 7.9	67.7, 9.9	54.3, 5.9	58.8, 9.4
BMI (kg/m^2^)	22.7, 2.0	24.6, 3.1	21.0, 2.03	24.8, 3.5
Upper arm circumference (cm)	28.6, 2.5	28.1, 3.1	25.9, 2.3	28.0, 3.4
Total fat mass BIA (kg)	13.7, 4.5	18.9, 5.9	15.2, 4.0	21.8, 6.3
Total fat mass DXA (kg)	14.7, 5.2	18.5, 6.3*	16.4, 4.1	20.3, 5.9*
Total body fat percentage BIA (%)	20.1, 5.1	27.5, 5.9	27.8, 5.1	36.2, 6.3
Total body fat percentage DXA (%)	22.1, 6.2	27.8, 6.7*	30.7, 5.2	36.0, 6.1*
ASM BIA (kg)	22.9, 2.6	20.5, 3.0	15.7, 1.8	14.6, 2.3
ASM DXA (kg)	22.2, 2.6	19.8, 2.8*	14.9, 1.7	14.3, 2.1*
ASMI BIA (kg/m^2^)	7.68, 0.54	7.44, 0.73	6.07, 0.48	6.11, 0.70
ASMI DXA (kg/m^2^)	7.47, 0.68	7.21, 0.83*	5.77, 0.53	6.04, 0.72*
Dominant hand grip (kg)	43.3, 6.7	34.5, 6.8	26.5, 3.8	22.3, 4.5
Gait speed (m/s)	1.61, 0.28	1.27, 0.26	1.49, 0.26	1.22, 0.26

*Data are mean ± SD. BMI, body mass index; ASM, appendicular skeletal muscle mass; ASMI, appendicular skeletal muscle mass index; BIA, bioelectrical impedance analysis; DXA, dual-energy X-ray absorptiometry*.

*A total of 10% of the older subjects were selected randomly to receive a measurement of muscle mass using the DXA method (n = 147 for men and n = 143 for women).

As shown in Table 2, by using existing definitions (7–12), the prevalence of sarcopenia in older adults ranged from 0 to 27.0% in men and 0 to 25.2% in women. The prevalence of low muscle mass using DXA varied from 0.7 to 51.0% in men and 16.1 to 30.1% in women, while the prevalence of low muscle mass using BIA was from 26.2 to 97.4% in men and 18.1 to 68.0% in women. Low handgrip ranged from 9.5 to 34% in men and 7.3 to 30.1% in women. The prevalence of low gait speed varied from 2.6 to 17.5% in men and 3.5 to 18.3% in women. Comparing the BIA and DXA, the prevalence of low muscle mass was significantly different in men in all the guidelines but significantly different in women only for the EWGSOP and AWGS cutoffs (Supplementary Table S2). Furthermore, the prevalence of low muscle mass was expectedly higher using the cutoffs defined in EWGSOP compared with other defined guidelines since it used higher thresholds for low handgrip and low ASMI using DXA and BIA.

**Table 2:** Sarcopenia prevalence based on different existing definitions.

**Existing definitions of sarcopenia**	**Prevalence**			**Prevalence**		
	**(DXA cohort**, ***n*** **= 290)**			**(BIA cohort**, ***n*** **= 2,821)**		
	**Male**	**Female**	***p*** **value**	**Male**	**Female**	***p*** **value**
	**(*n* = 147)**	**(*n* = 143)**		**(*n* = 1,398)**	**(*n* = 1,423)**	
**AWGS (** **11** **)**	9 (6.1)	7 (4.9)	0.65	84 (6.0)	123 (8.6)	0.007
Low muscle mass	62 (42.2)	37 (25.9)	0.003	366 (26.2)	391 (27.5)	0.44
Low hand grip	14 (9.5)	26 (18.2)	0.03	141 (10.1)	226 (15.9)	<0.001
Low gait speed	4 (2.7)	5 (3.5)	0.70	38 (2.7)	60 (4.2)	0.03
**IWGS (** **7** **)**	12 (8.2)	5 (3.5)	0.09	93 (6.7)	102 (7.2)	0.59
Low muscle mass	75 (51.0)	43 (30.1)	<0.001	538 (38.5)	369 (25.9)	<0.001
Low hand grip	.	.	.	.	.	.
Low gait speed	20 (13.6)	20 (14.0)	0.93	189 (13.5)	260 (18.3)	0.001
**EWGSOP (** **8** **)**	33 (22.4)	14 (9.8)	0.003	378 (27.0)	359 (25.2)	0.27
Low muscle mass	75 (51.0)	43 (30.1)	<0.001	1362 (97.4)	968 (68.0)	<0.0001
Low hand grip	50 (34.0)	43 (30.1)	0.47	371 (26.5)	425 (29.9)	0.05
Low gait speed	4 (2.7)	5 (3.5)	0.70	37 (2.6)	55 (3.9)	0.07
**FNIH (** **9** **)**	2 (1.4)	0 (0)	0.16	11 (0.8)	12 (0.8)	0.87
Low muscle mass	73 (49.7)	23 (16.1)	<0.001	490 (35.1)	257 (18.1)	<0.001
Low hand grip	14 (9.5)	11 (7.7)	0.58	141 (10.1)	104 (7.3)	0.01
Low gait speed	4 (2.7)	5 (3.5)	0.70	38 (2.7)	60 (4.2)	0.03
**AWGS (** **32** **)**	23 (15.6)	7 (4.9)	0.003	155 (11.1)	171 (12.0)	0.44
Low muscle mass	62 (42.2)	29 (20.3)	<0.0001	366 (26.2)	391 (27.5)	0.44
Low hand grip	35 (23.8)	26 (18.2)	0.24	189 (13.5)	260 (18.3)	<0.001
Low gait speed	20 (13.6)	20 (14.0)	0.93	244 (17.5)	226 (15.9)	0.26
**EWGSOP2 (** **10** **)**	0 (0.0)	2 (1.4)	0.15	93 (6.7)	44 (3.1)	<0.001
Low muscle mass	1 (0.7)	37 (25.9)	<0.001	366 (26.2)	262 (18.4)	<0.001
Low hand grip	22 (15.0)	11 (7.7)	0.05	180 (12.9)	104 (7.3)	<0.001
Low gait speed	4 (2.7)	5 (3.5)	0.70	38 (2.7)	60 (4.2)	0.03

*Data represented n (%). DEXA, dual-energy X-ray absorptiometry; BIA, bioelectrical impedance analysis; AWGS, Asia Working Group of Sarcopenia; IWGS, International working group on sarcopenia; EWGSOP, European Working Group on Sarcopenia in Older People; FNIH, Foundation for the National Institute of Health*.

### Identifying Optimal BIA Cutoffs for Low ASMI According to DXA Cutoffs

Dual-energy X-ray absorptiometry results showed (Supplementary Table S1) that the three cutoff points used for men were 6.10 kg/m^2^ (ASMI-2 SD), 6.79 kg/m^2^ (ASMI-1 SD), and 6.53 kg/m^2^ (ASMI 20) and in women, the cutoff points were 4.71, 5.24, and 5.40 kg/m^2^, respectively. Accordingly, the proportions of low ASMI in older subjects were 10.2% (ASMI-2 SD), 30.6% (ASMI-1 SD), and 21.1% (ASMI 20) in men and 2.8% (ASMI-2 SD), 14.0% (ASMI-1 SD), and 20.3% (ASMI 20) in women. The prevalence defined by ASMI-2 SD and ASMI-1 SD showed a significant gender difference (*p* = 0.01 and *p* = 0.007, respectively). Consequently, 6.53 kg/m^2^ in men and 5.40 kg/m^2^ in women were selected as the best cutoff values, respectively, determined by the 20th percentiles of the older adults' group as shown in Table 3.

**Table 3:** Percentiles for muscle mass, handgrip strength, and calf circumference parameters of the older reference population (*n* = 2,821).

**Percentiles**	**Males (*****n*** **= 1398)**				**Females (*****n*** **= 1423)**			
	ASMI-DEXA*	**ASMI-BIA**	**Hand grip**	**Gait speed**	ASMI-DEXA*	**ASMI-BIA**	**Hand grip**	**Gait speed**
	**(kg/m^**2**^)**	**(kg/m^**2**^)**	**(kg)**	**(m/s)**	**(kg/m^**2**^)**	**(kg/m^**2**^)**	**(kg)**	**(m/s)**
5	6.00	6.29	23.9	0.89	4.93	5.02	15.0	0.83
10	6.10	6.57	25.9	0.96	5.03	5.28	16.6	0.91
15	6.37	6.72	27.5	1.01	5.28	5.42	17.7	0.97
20	6.53	6.88	28.5	1.05	5.40	5.55	18.6	1.01
25	6.59	6.97	29.7	1.1	5.46	5.66	19.3	1.04
30	6.76	7.07	30.7	1.14	5.66	5.76	20.0	1.08
35	6.88	7.16	31.8	1.16	5.77	5.86	20.6	1.11
40	6.98	7.26	32.6	1.2	5.86	5.94	21.2	1.14
45	7.06	7.33	33.5	1.22	6.00	6.03	21.8	1.17
50	7.20	7.43	34.3	1.26	6.10	6.09	22.3	1.19
55	7.31	7.51	35.2	1.29	6.15	6.18	22.9	1.23
60	7.40	7.60	36.1	1.32	6.25	6.27	23.4	1.26
65	7.50	7.71	37.1	1.35	6.33	6.38	24.0	1.30
70	7.55	7.81	38.1	1.39	6.43	6.46	24.7	1.34
75	7.66	7.93	39	1.42	6.57	6.54	25.2	1.38
80	7.79	8.04	40.3	1.45	6.67	6.66	26.2	1.43
85	7.99	8.19	41.8	1.52	6.80	6.77	27.1	1.47
90	8.29	8.35	43.3	1.59	6.98	6.95	28.1	1.55
95	8.73	8.64	46	1.69	7.10	7.20	29.5	1.68

*ASMI, appendicular skeletal muscle mass index; BIA, bioelectrical impedance analysis; DXA, dual-energy X-ray absorptiometry*.

*A total of 10% of the older subjects were selected randomly to receive a measurement of muscle mass using the DXA method (n = 147 for men and n = 143 for women).

**Figure 1:**
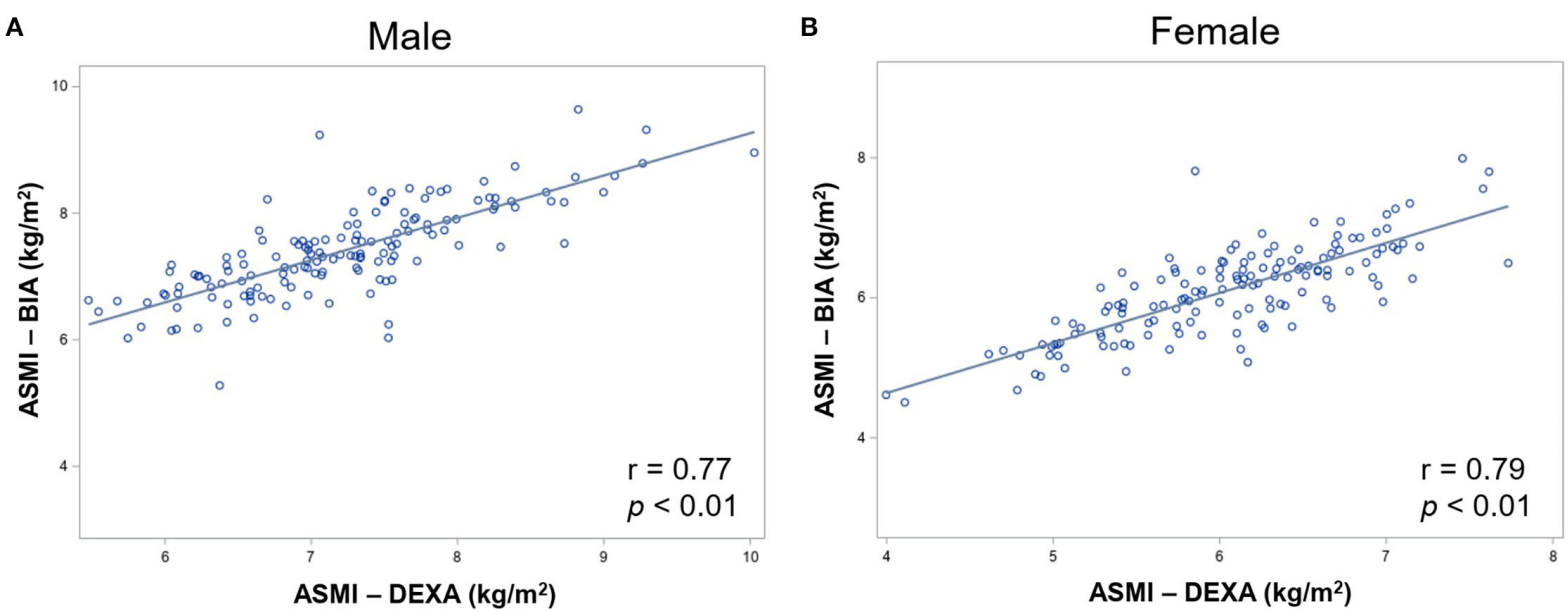
Correlation between DXA and BIA measured ASM in **(A)** male and **(B)** female participants. DXA, dual-energy X-ray absorptiometry; BIA, bioelectrical impedance analysis; ASMI, appendicular skeletal muscle mass.

**Figure 2:**
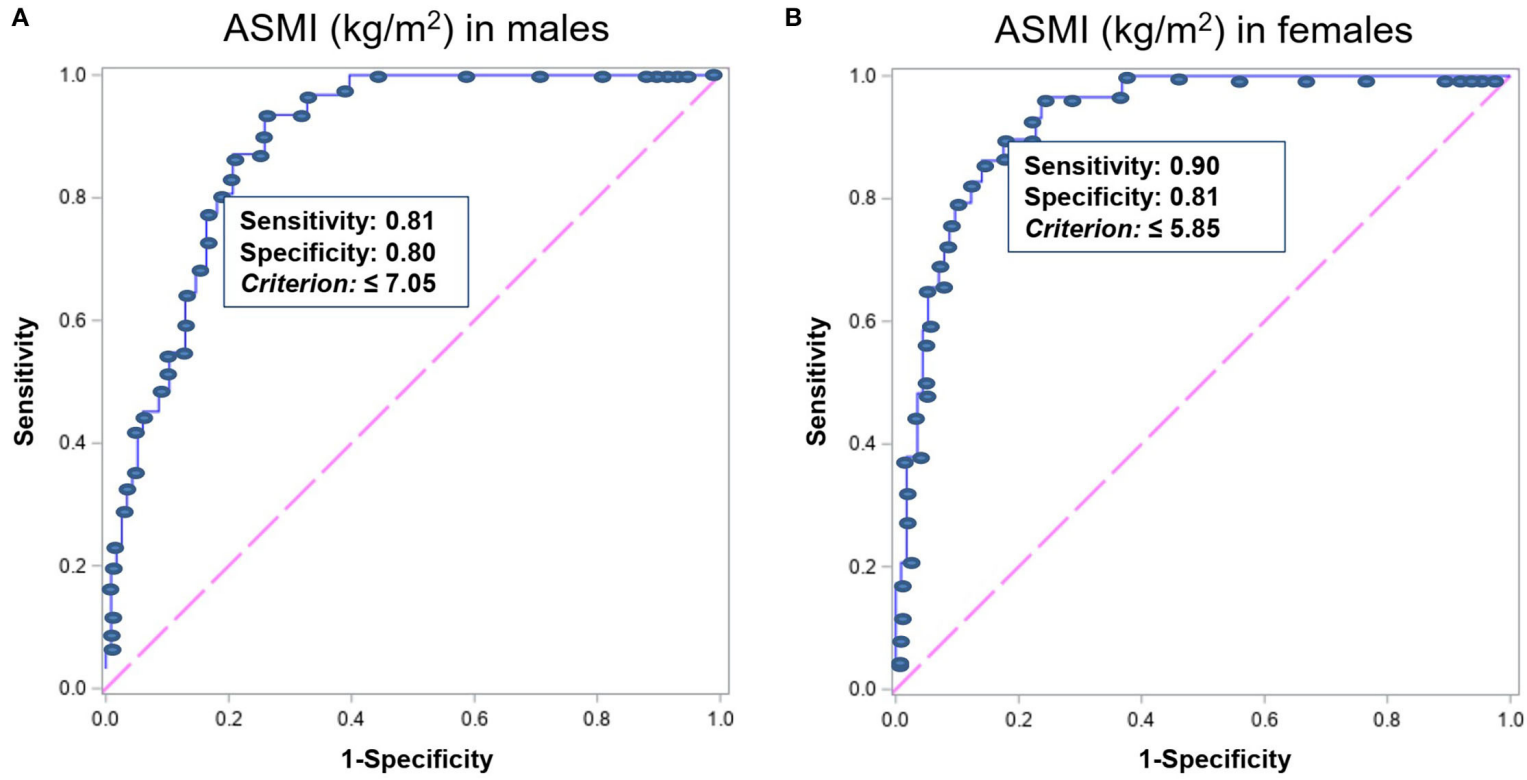
The ROC analysis results of ASMI measured by BIA for predicting ASMI measured by DXA <6.53 and 5.40 kg/m^2^ in men and women, respectively, indicating the ASMI value of ideal highest sensitivity and specificity in **(A)** men (*n* = 1,398) and **(B)** women (*n* = 1,423). ROC, receiver operating characteristics; ASMI, appendicular skeletal muscle mass index; BIA, bioelectrical impedance analysis; DXA, dual-energy X-ray absorptiometry.

Dual-energy X-ray absorptiometry results were considered as criteria to validate BIA measurements. The ASMI measured by BIA showed strong correlations with the measurements by DXA in both men and women (*r* = 0.77 and *p* < 0.001 in men; *r* = 0.79 and *p* < 0.001 in women; Figures 1A,B). Bland–Altman plot (Supplementary Figure S1) shows good agreement between the BIA and DXA measurements of ASMI. Additionally, the concordance correlation coefficient also showed substantial agreement between BIA and DXA on ASMI for males, 95% CI, 0.83–0.94; for females, 95% CI, 0.90–0.97. The AUCs of the low muscle mass index measured by BIA were 0.89 (95% CI, 0.97–0.98) and 0.93 (95% CI, 0.94–0.97) in men and women, respectively. The low ASMI value using BIA that best predicts the low ASMI using DXA (<6.53 and <5.40 kg/m^2^ in men and women, respectively) was <7.05 kg/m^2^ in men (sensitivity: 0.81, 95% CI: 0.63–0.93; specificity: 0.80, 95% CI: 0.72–0.87) and <5.85 kg/m^2^ in women (sensitivity: 0.90, 95% CI: 0.73–0.98; specificity: 0.81, 95% CI: 0.72–0.87) (Figures 2A,B).

### Cutoffs of Muscle Strength and Gait Speed

Percentiles of muscle mass, handgrip strength, and gait speed are given in Table 3. Following the AMI rules measured by DXA, the lowest 20th percentile value of 28.5 and 18.6 kg in men and women, respectively, from the older adults' group was selected as cutoffs for low handgrip strength. In addition, 1.05 and 1.01 m/s in men and women were, respectively, selected for low gait speed based on the lowest 20% of the older adults' group.

Based on the derived cutoffs, 14.2% of men and 15.7% of women in the older Chinese study population were classified to have sarcopenia (Table 4). The prevalence was 7.5 and 10.1% (60–69 years), 24.2 and 24.5% (70–79 years), and 43.6 and 62.5% (≥80 years) in men and women, respectively.

**Table 4:** Prevalence of low muscle mass, low hand strength, low gait speed, and sarcopenia by gender-based on proposed cutoffs.

**Parameters**	**Proposed cutoffs**	**Overall**	**60 y−69 y**	**70 y−79 y**	**≥80 y**
**Male**		**N = 1,398**	**N=904**	**N = 439**	**N = 55**
Low muscle mass (BIA), kg/m	7.05	407 (29.1)	209 (23.1)	169 (38.5)	29 (52.7)
Low hand grip, kg	28.5	278 (19.9)	102 (11.3)	141 (32.1)	35 (63.6)
Low gait speed, m/s	1.05	258 (18.5)	116 (12.8)	116 (26.4)	26 (47.2)
Sarcopenia	+ or +	198 (14.2)	68 (7.5)	106 (24.2)	24 (43.6)
**Female**		***N*** **= 1,423**	***N*** **= 979**	***N*** **= 404**	***N*** **= 40**
Low muscle mass (BIA), kg/m	5.85	485 (34.1)	279 (28.5)	180 (44.6)	26 (65.0)
Low hand grip, kg	18.6	283 (19.9)	135 (13.8)	118 (29.2)	30 (75.0)
Low gait speed, m/s	1.01	276 (19.4)	137 (14.0)	112 (27.7)	27 (67.5)
Sarcopenia	+ or +	223 (15.7)	99 (10.1)	99 (24.5)	25 (62.5)

*Values were n (%). BIA, bioelectrical impedance analysis*.

### Comparison With Existing Guidelines

Sensitivity, specificity, negative predictive values, AUC, and accuracy were high between the proposed cutoffs derived in this study and the AWGS, IWGS, and EWGSOP2 guidelines, but lower relative to FNIH and EWGSOP. This may be due to the different low muscle mass measures used as follows: ASMI for our cutoffs, ASM/BMI for FNIH, the requirement to meet low muscle mass, low grip strength, and low gait speed with the FNIH, and the higher thresholds for EWGSOP. The low positive predictive values, as well as the lower values for women relative to men, were expected since our proposed cutoffs, and more so for women than men, tended to be higher than existing guidelines (Table 5). Cutoffs for sarcopenia and its components as defined by existing guidelines used in the comparisons are provided in Supplementary Table S3.

**Table 5:** Sensitivity, specificity, positive and negative predictive values, area under the curve (AUC), and accuracy between the proposed cutoffs relative to existing guidelines.

**Reference guideline**	**Sensitivity (%)**	**Specificity (%)**	**Positive predictive value (%)**	**Negative predictive value (%)**	**Accuracy (%)**	**Area under curve**
**AWGS (** **11** **)**						
Male	100	91.3	41.9	100	91.8	0.9215
Female	100	72.4	25.5	100	74.8	0.9209
**AWGS (** **32** **)**						
Male	100	96.5	78.3	100	96.9	0.9357
Female	100	75.2	35.5	100	78.1	0.9317
**IWGS (** **7** **)**						
Male	84.9	90.9	39.9	98.8	90.5	0.8711
Female	100	71.2	21.2	100	73.3	0.9140
**EWGSOP (** **8** **)**						
Male	42.3	96.3	80.8	81.8	81.7	0.6901
Female	65.5	76.8	48.8	86.8	73.9	0.8072
**EWGSOP2 (** **10** **)**						
Male	100	92.0	47.0	100	92.5	0.9267
Female	100	68.2	9.1	100	69.2	0.9400
**FNIH (** **9** **)**						
Male	54.5	86.2	3.0	99.6	85.9	0.6914
Female	72.7	66.4	1.7	99.7	66.5	0.7641

*Data represented %. AWGS, Asia Working Group on Sarcopenia; IWGS, International working group on sarcopenia; EWGSOP, European Working Group on Sarcopenia in Older People; FNIH, Foundation for the National Institute of Health*.

## Discussion

This study establishes cutoffs of grip strength, gait speed, and BIA-derived low muscle mass based on a large, gender- and geography-diverse Chinese study population. We particularly recruited equal numbers of male and female participants in their early adult years in order to capture peak skeletal muscle mass in both genders since muscle mass gradually declined with aging. We also specifically chose Beijing and Shanghai as study sites since they were two of the largest cities in China representing distinguishable diets and lifestyles in Northern and Eastern China. Our results confirm that BIA provides a valid and easy estimation of skeletal muscle mass using DEXA as a reference standard. The findings of our study contribute new evidence to a better understanding of muscle quantity, muscle function, and performance in healthy young Chinese adults and older Chinese adults. These values can be potentially used for assessing sarcopenia in older Chinese people in an epidemiological and clinical setting.

Obvious differences exist in the cutoffs among the proposed of this study and the AWGS, EWGSOP, EWGSOP2, IWGS, and FNIH recommendations. The cutoff values derived from the Caucasian Panel criteria based on different racial reference populations are inapplicable to the Chinese population. Besides, data from the Chinese population residing in mainland China are lacking in the AWGS. In this large cohort, the findings first demonstrated how existing sarcopenia definitions impact the proportion of sarcopenia in the Chinese older population. With the exception of EWGSOP, the prevalence of sarcopenia in the older study population ranged from 0.0 to 15.6% in men and 0.0 to 12.0% in women with significant gender differences. It is lower than most of the reported prevalence ranging from 10 to 40% (2, 22, 26–30). It is worth noting that existing sarcopenia definitions produced underestimated prevalence of sarcopenia. It decreases awareness of sarcopenia and its risk in China. Underestimating sarcopenia in older Chinese adults is not conducive to early prevention.

Older and younger adults show differences in weight, height, muscle mass, muscle strength, and physical performance due to the aging process, which was also observed in our study (Table 1) but excluded the ASMI in women. The mean of ASMI is similar or even higher in older female adults than in the young group in this study (ASMI BIA: 6.07 kg/m^2^ in young women vs. 6.11 kg/m^2^ in older women; ASMI DXA: 5.77 kg/m^2^ in young women vs. 6.04 kg/m^2^ in older women). That is the reason why 4.71 kg/m^2^ (ASMI-2 SD) and 5.24 kg/m^2^ (ASMI-1 SD) in young adults are even lower than 5.40 kg/m^2^ (ASMI 20) in the older women in this study. Consequently, this produced an unacceptably low proportion of low muscle mass in older women (2.8 and 14.0% for ASMI-2 SD and ASMI-1 SD, respectively). Interestingly, other studies in Asian cohorts reported a similar result (33). This may be due to Asian women in their 20 s and 30 s tending to prefer a slim body with a focus on diet restriction in the latest decades, which harms muscle development. Besides, the prevalence defined by ASMI-2 SD and ASMI-1 SD showed significant gender difference (*p* = 0.01 and *p* = 0.007, respectively). Therefore, the lowest 20% of the older group is the optimal standard cutoff value for DXA (6.53 and 5.40 kg/m^2^ in men and women, respectively) to obtain a meaningful proportion of low muscle mass without significant gender difference.

Dual-energy X-ray absorptiometry is slowly becoming more available even if it remains too costly and requires expertise in performing measurements. The application of the BIA method in clinical practice used to be criticized due to its large inaccuracy resulting from analysis assumptions related to body shapes and the distribution of current density (34). However, recent advances in technology facilitate the development of new BIA devices with improved accuracy and high precision (35). The findings of this study showed excellent agreement between BIA and DXA methods in muscle mass measurement, though a small systematic bias existed. The reported systematic bias in this study is within the range of previously published data (0.1–2.1 kg) (18, 36, 37). Considering that the DXA used in estimating body composition is the reference method, the low ASMI threshold of the BIA using the ROC analysis represented the cutoff value of <6.53 and 5.40 kg/m^2^ in men and women, respectively, to identify the ASMI of the participants measured by BIA. The data in this study indicated that the low ASMI measured by BIA cut-point thresholds are 7.05 kg/m^2^ (sensitivity, 0.81; specificity, 0.80) in men and 5.85 kg/m^2^ (sensitivity, 0.90; specificity, 0.81) in women. This finding follows the data of sarcopenia assessment in Korea (38), which is very important for assessing and managing sarcopenia in China. Among three components, the methods to determine low muscle mass using DXA show limitations in application in primary care settings. Therefore, our study replaces DXA with BIA, an easier but accurate measure, which will enhance the screening of sarcopenia in the general population, to achieve attention and early intervention. We proposed the cutoffs, 7.05 kg/m^2^ for men, and 5.85 kg/m^2^ for women, showing high sensitivity of over 80% to identify older people with reduced muscle mass.

The cutoff of muscle strength for IWGS, FNIH, EWGSOP, and EWGSOP2 (7–10) showed a significant difference in low muscle strength proportion between men and women. In this study, the cutoff for handgrip strength was found to be 28.5 and 18.6 kg in men and women, using the sex-specific lowest 20% quintile points of the handgrip strength in the older study population, respectively. It is the optimal cutoff of muscle strength for older adults in China. The finding is similar to the cutoff of low handgrip strength (28.8 and 18.2 kg for men and women, respectively) in sarcopenia definition in Japan (39). Of note, 0.8 m/s is the cutoff for low gait speed used in EWGSOP, FNIH, and AWGS (8, 9, 11). But it showed an extremely low proportion (2.7–4.2%) of low gait speed without clinical relevance since 0.8 m/s is slower than the lowest 5% gait speed in the older male and female study population (Table 2). Therefore, the same rule using the sex-specific lowest 20% in the older study group was followed to define the thresholds for gait speed. The corresponding thresholds for gait speed were 1.05 and 1.01 m/s for men and women, respectively. By using the new cutoff thresholds of three components set up in this study, the overall proportion of sarcopenia was 14.2 and 15.7% for men and women, respectively. This new threshold to be used for assessing sarcopenia in older Chinese people is more optimal than other existing sarcopenia definitions.

This study has several strengths. First, this study had a relatively large sample size of more than 2,800 and 400 older and young Chinese people, respectively. Both the older and young study groups have sample sizes higher than or comparable with most of the other reference population data in the literature (21, 24, 26, 32, 39, 40). Second, this study selected three cities (i.e., Beijing, Shanghai, and Chengdu) according to the characteristics of the typical life and dietary pattern to ensure the representativeness of the study population. Finally, by using DXA as the gold standard technology for the assessment of skeletal muscle, this study proposed cutoff thresholds for BIA as a good portable alternative measure to DXA. This will improve the application of screening and awareness of sarcopenia in the older Chinese population. However, it also has several limitations. The first limitation of this study is that the study population did not include the rural population. Thus, care should be exercised when extrapolating these cutoffs to different populations. Second, although we chose residents of three representative cities in mainland China as the study population for this study, it has to be acknowledged that there may be some potential selection bias in the sample, so in future studies, we will further develop a variety of new joint surveys to increase the generalizability and persuasiveness of the findings. The third limitation is we did not design a longitudinal follow-up of the cohort to reveal the changes in muscle strength, muscle mass, and physical performance over time.

In conclusion, this study proposed the sex-specific cutoff for three components of sarcopenia. The 7.05 kg/m^2^ in men and 5.85 kg/m^2^ in women were selected as the optimal cutoffs of BIA for low ASMI. The 28.5 kg and 1.05 m/s for men and 18.6 kg and 1.01 m/s for women were determined as the optimal cutoffs for handgrip strength and gait speed, respectively. These optimal cutoffs will enhance practicability for screening sarcopenia in primary care and clinical settings.

## Data Availability Statement

The original contributions presented in the study are included in the article/Supplementary Material, further inquiries can be directed to the corresponding author/s.

## Ethics Statement

The studies involving human participants were reviewed and approved by the Institutional Review Board of Peking Union Medical College Hospital. The patients/participants provided their written informed consent to participate in this study.

## Author Contributions

WC, SG, and BD contributed to the conception and design of the research. WC, SG, BD, XF, HW, SH, XS, WS, AM, TL, and HL contributed to the acquisition of the data. QD, VP, GB, and MY contributed to the analysis and interpretation of the data. QD and SG drafted the manuscript. QD substantially revised the submitted version. All authors critically revised the manuscript, agreed to be fully accountable for ensuring the integrity and accuracy of the work, and read and approved the final manuscript.

## Funding

This study was supported by Abbott Nutrition Research and Development, Peking Union Medical College Hospital, Sixth People's Hospital Affiliated Shanghai Jiao Tong University, West China Hospital Affiliated to Sichuan University and Abbott Nutrition Research and Development were involved in the study design, data collection and analysis, or preparation of the manuscript. Cognizant Technology Solution Pvt., Ltd., provided only statistical services to Abbott Nutrition. This study was supported by grants from the Abbott Laboratories Scientific funding BL22/23.

## Conflict of Interest

VP and MY are employees of Cognizant Technology Solutions, which provides statistical services to Abbott. The remaining authors declare that the research was conducted in the absence of any commercial or financial relationships that could be construed as a potential conflict of interest.

## Publisher's Note

All claims expressed in this article are solely those of the authors and do not necessarily represent those of their affiliated organizations, or those of the publisher, the editors and the reviewers. Any product that may be evaluated in this article, or claim that may be made by its manufacturer, is not guaranteed or endorsed by the publisher.

## Supplementary Material

Supplementary Figure S1 Bland–Altman plot of ASMI between BIA and DXA. ASMI, appendicular skeletal muscle mass index; BIA, bioelectrical impedance analysis; DXA, dual-energy X-ray absorptiometry.

Supplementary Table S1 Cutoff values and proportions of flow muscle mass based on DXA.

Supplementary Table S2 Comparison of prevalence's between BIA and DXA.

Supplementary Table S3 Cutoff's for sarcopenia based on different guidelines.
