# Supplementary material for: Optimal Cutoffs for the Diagnosis of Sarcopenia in Older Chinese Adults
Source: Front Nutr. 2022 Jul 5;9:853323. doi: 10.3389/fnut.2022.853323 (PMC9294727; doi:10.3389/fnut.2022.853323)
Supplement: Supplementary Figure S1 — Bland–Altman plot of ASMI between BIA and DXA. ASMI, appendicular skeletal muscle mass index; BIA, bioelectrical impedance analysis; DXA, dual-energy X-ray absorptiometry. [file Data_Sheet_1.zip › Table S1.PDF]

Table S1 Cutoff values and proportions of low muscle mass based on DXA

Gender specific cutoff values			Male (N=147)	Female (N=143)	Gender difference *p*
	Male	Female			
ASMI-2 SD, kg/m^2	6.10	4.71	15 (10.2)	4(2.8)	0.01
ASMI-1 SD, kg/m^2	6.79	5.24	45(30.6)	20(14.0)	0.007
ASMI P20, kg/m^2	6.53	5.40	31(21.1)	29(20.3)	0.87

Data of low muscle mass prevalence represented n(%). ASMI, appendicular skeletal muscle mass index; SD, standard deviation. The prevalence of low muscle mass between the gender was compared using the Chi-square test or Fishers exact. During testing, $P<0.05$ was considered to be statistically significant.
