# Supplementary material for: Optimal Cutoffs for the Diagnosis of Sarcopenia in Older Chinese Adults
Source: Front Nutr. 2022 Jul 5;9:853323. doi: 10.3389/fnut.2022.853323 (PMC9294727; doi:10.3389/fnut.2022.853323)
Supplement: Supplementary Figure S1 — Bland–Altman plot of ASMI between BIA and DXA. ASMI, appendicular skeletal muscle mass index; BIA, bioelectrical impedance analysis; DXA, dual-energy X-ray absorptiometry. [file Data_Sheet_1.zip › Table S2.DOCX]

Table S2: Comparison of prevalence’s between BIA and DXA

| **Definitions of Sarcopenia** | **Prevalence** | | | | | | | |  |  |  |
| --- | --- | --- | --- | --- | --- | --- | --- | --- | --- | --- | --- |
|  |  |  |  |  |  |  |  |  |  | |  |
|  |  | **Male (n=147)** | | |  | **Female (n=143)** | | | |  | |
|  |  |  |  |  |  |  |  |  |  |  | |
|  |  | **BIA** | **DEXA** | ***p* value** |  | **BIA** | **DEXA** | ***p* value** | |  | |
| AWGS[11] | Sarcopenia | 6(4.1) | 9(6.1) | 0.08 |  | 10(7.0) | 7(4.9) | 0.08 | |  | |
|  | Low muscle mass | 41(27.9) | 62(42.2) | <0.001 |  | 42(29.4) | 37(25.9) | 0.3 | |  | |
| IWGS[7] | Sarcopenia | 11(7.5) | 12(8.2) | 0.56 |  | 3(2.1) | 5(3.5) | 0.32 | |  | |
|  | Low muscle mass | 60(40.8) | 75(51.0) | 0.01 |  | 40(28.0) | 43(30.1) | 0.55 | |  | |
| EWGSOP[8] | Sarcopenia | 50(34.0) | 33 (22.4) | <0.001 |  | 37(25.9) | 14(9.8) | <0.001 | |  | |
|  | Low muscle mass | 143(97.3) | 75(51.0) | <0.001 |  | 101(70.6) | 43(30.1) | <0.001 | |  | |
| AWGS[12] | Sarcopenia | 15(10.2) | 23(15.7) | 0.03 |  | 12(8.4) | 7(4.9) | 0.03 | |  | |
|  | Low muscle mass | 41(27.9) | 62(42.2) | <0.001 |  | 42(29.4) | 29(20.3) | 0.005 | |  | |
| EWGSOP2[10] | Sarcopenia | 0(0.0) | 0(0.0) | NA |  | 2(1.4) | 2(1.4) | 1 | |  | |
|  | Low muscle mass | 41(27.9) | 1(0.7) | <0.001 |  | 31(21.7) | 37(25.9) | 0.18 | |  | |
| FNIH[9] | Sarcopenia | 2(1.4) | 2(1.4) | NA |  | 0(0.0) | 0(0.0) | NA | |  | |
|  | Low muscle mass | 62(42.2) | 73(49.7) | 0.04 |  | 24(16.8) | 23(16.1) | 0.8 | |  | |

Data represented n(%). DEXA, dual-energy x-ray absorptiometry; BIA, bioelectrical impedance analysis; AWGS, Asia Working Group of Sarcopenia; IWGS, International working group on sarcopenia; EWGSOP, European Working Group on Sarcopenia in Older People; FNIH, Foundation for the National Institute of Health. P-value is evaluated based on McNemar’s Test.
