# Supplementary material for: Optimal Cutoffs for the Diagnosis of Sarcopenia in Older Chinese Adults
Source: Front Nutr. 2022 Jul 5;9:853323. doi: 10.3389/fnut.2022.853323 (PMC9294727; doi:10.3389/fnut.2022.853323)
Supplement: Supplementary Figure S1 — Bland–Altman plot of ASMI between BIA and DXA. ASMI, appendicular skeletal muscle mass index; BIA, bioelectrical impedance analysis; DXA, dual-energy X-ray absorptiometry. [file Data_Sheet_1.zip › Table S3 .DOCX]

|  | **① Low muscle mass** | **② Low HS (kg)** | **③ Low GS (m/s)** | **Sarcopenia** |
| --- | --- | --- | --- | --- |
| **AWGS 2014 [11]** | Height adjusted skeletal muscle mass  DEXA  < 7.0 kg/m^2^ for Men  < 5.5 kg/m^2^ for Women   BIA  < 7.0 kg/m^2^ for Men.  < 5.7 kg/m^2^ for Women | < 26 kg for Men  < 18 kg Women | ≤ 0.8 m/s for both gender | ① + ② or ① + ③ |
| **IWGS (2009) [7]** | SMI  ≤ 7.23 kg/m2 for Men  ≤ 5.67 kg/m2 for Women |  | < 1.0 m/s for both genders | ① + ③ |
| **EWGSOP (2010) [8]** | SMI  DEXA (Health ABC)  ≤ 7.23 kg/m2 for Men  ≤ 5.67 kg/m2 for Women  BIA (using prediction equation)  ≤ 8.87 kg/m2 for Men.  ≤ 6.42 kg/m2 for Women | < 30 kg for Men  < 20 kg for Women | < 0.8 m/s for both gender | ① + ② or ① + ③ |
| **FNIH [9]** | ASM/BMI < 0.789 for Men  <0.512 for women | < 26 kg for Men  < 16 kg for women | ≤ 0.8 m/s for both genders | ① + ② + ③ |
| **EWGSOP2 (2019) [10]** | SMI  < 7.0 kg/m2 for Men  < 5.5 kg/m2 for Women | < 27 kg for Men  < 16 kg for women | ≤ 0.8 m/s for both genders | ① + ② -> sarcopenia  ① + ② + ③ -> severe sarcopenia |
| **AWGS (2019) [40]** | DEXA  < 7.0 kg/m2 for Men  < 5.4 kg/m2 for Women   BIA  < 7.0 kg/m2 for Men  < 5.7 kg/m2 for Women | < 28 kg for Men  < 18 kg for Women | < 1.0 m/s for both genders | ① + ② or ① + ③  ① + ② + ③ -> severe sarcopenia |

Table S3 : Cutoff’s for sarcopenia based on different guidelines
