# Supplementary figures and images for: Optimal Cutoffs for the Diagnosis of Sarcopenia in Older Chinese Adults
Source: Front Nutr. 2022 Jul 5;9:853323. doi: 10.3389/fnut.2022.853323 (PMC9294727; doi:10.3389/fnut.2022.853323)

**Figure S1. Bland-Altman plot of ASMI between BIA and DXA**

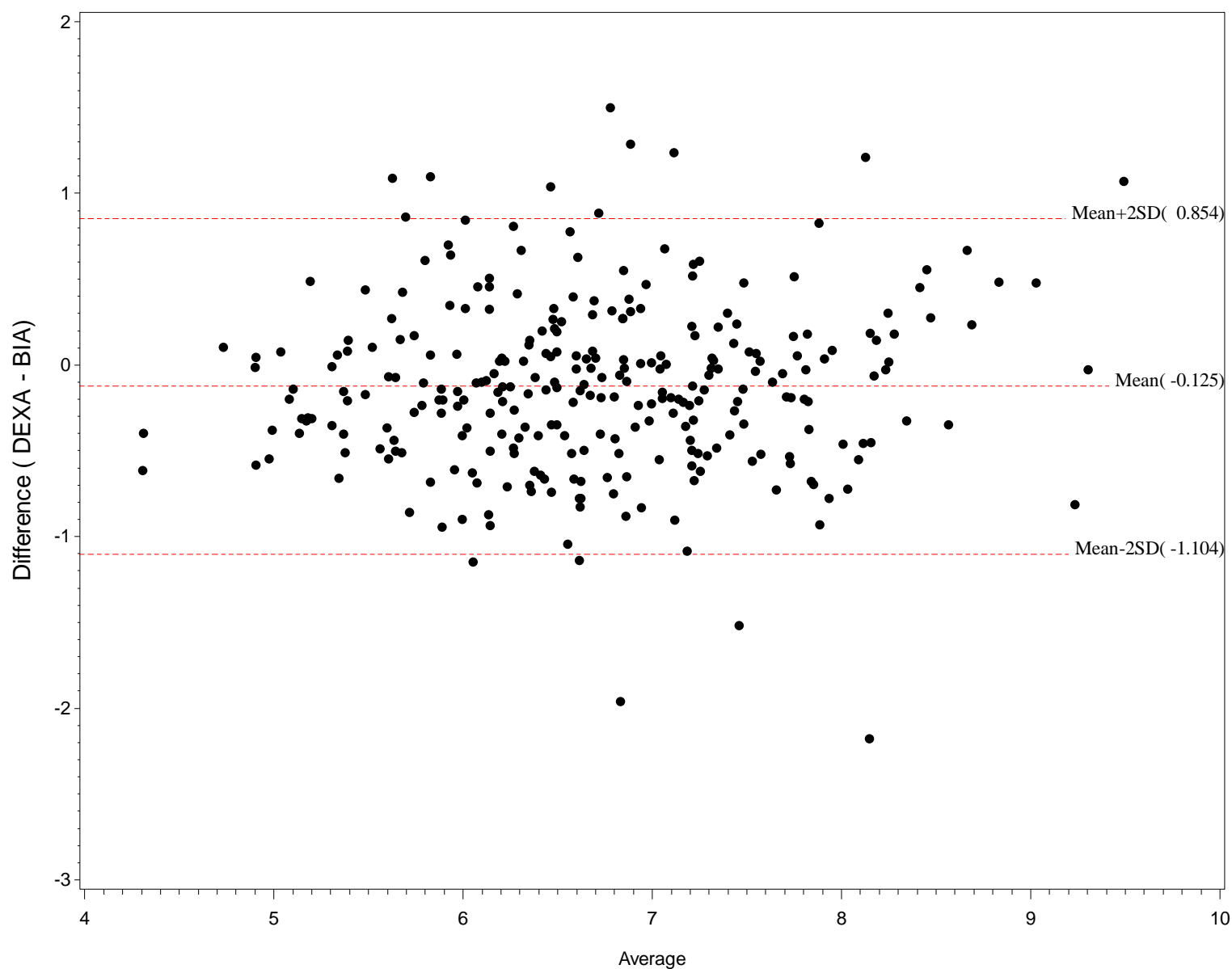

Supplement: Supplementary Figure S1 — Bland–Altman plot of ASMI between BIA and DXA. ASMI, appendicular skeletal muscle mass index; BIA, bioelectrical impedance analysis; DXA, dual-energy X-ray absorptiometry. [file Data_Sheet_1.zip › Figure S1.PDF]
